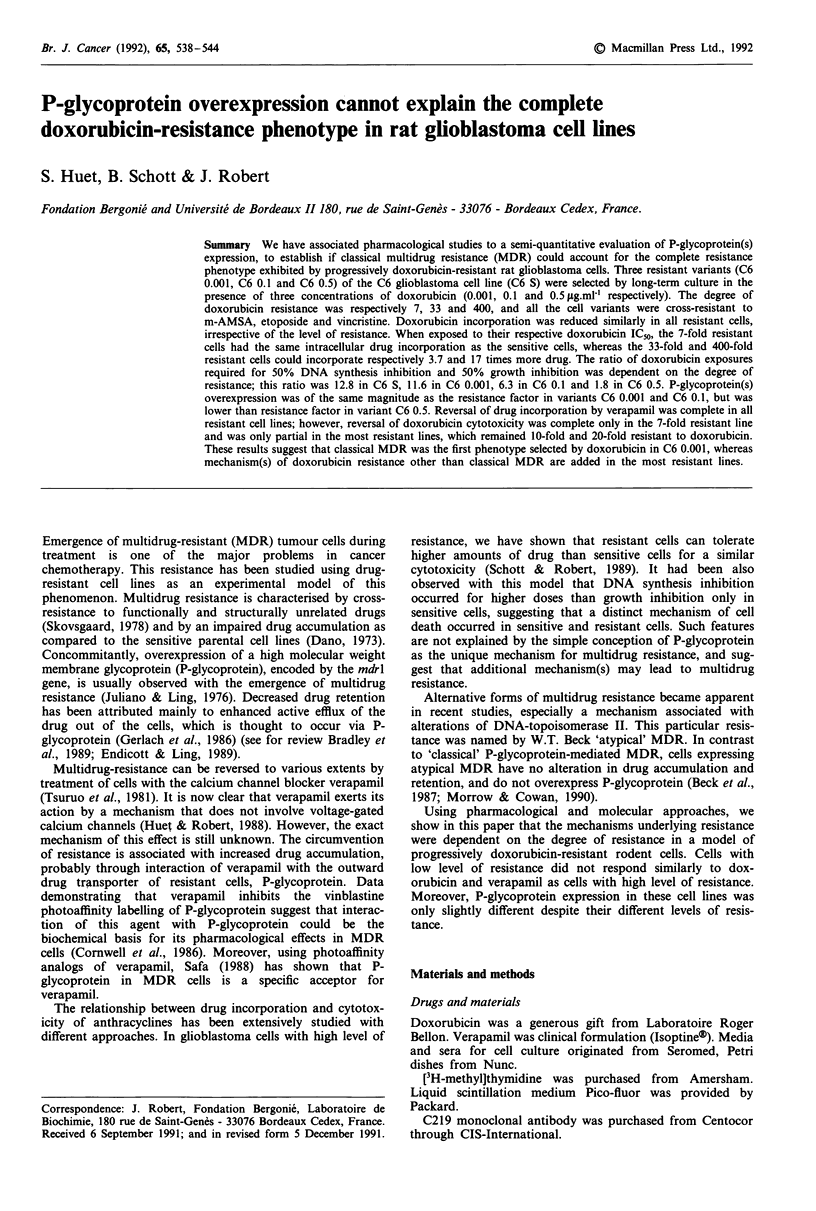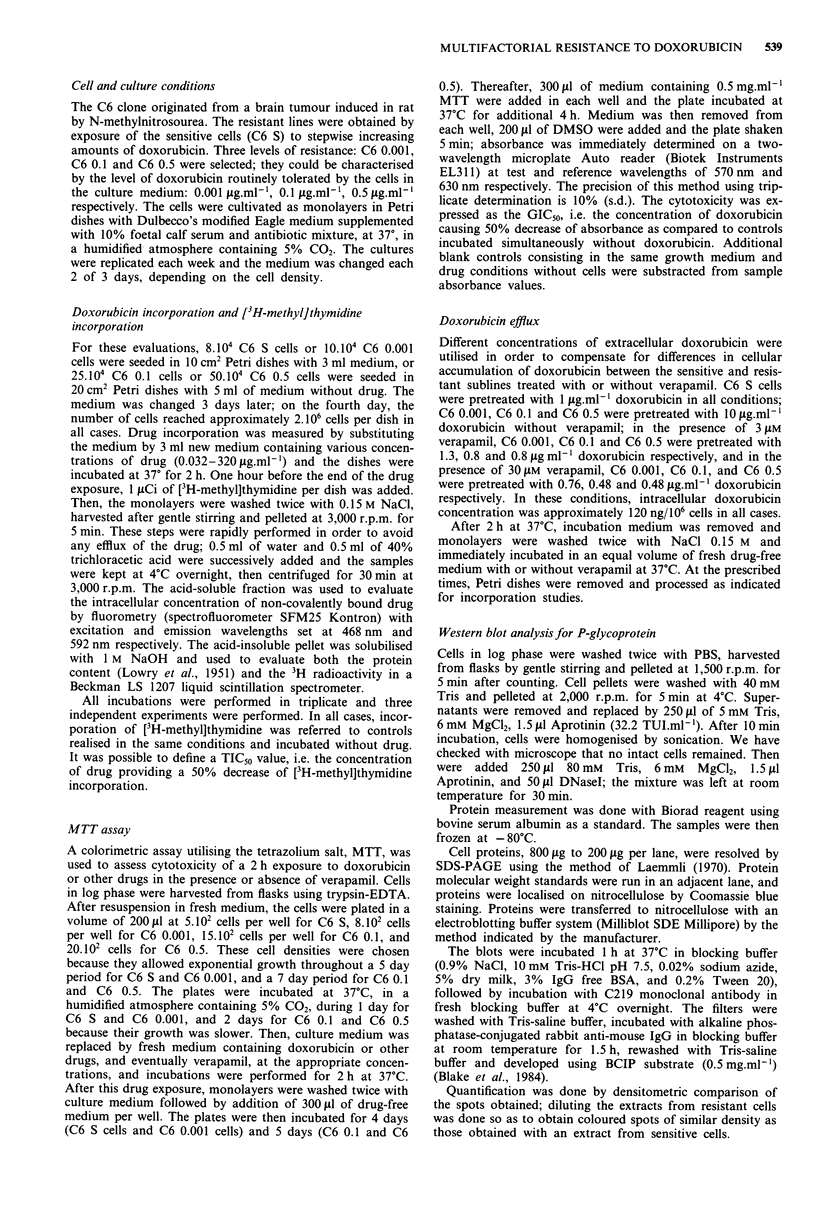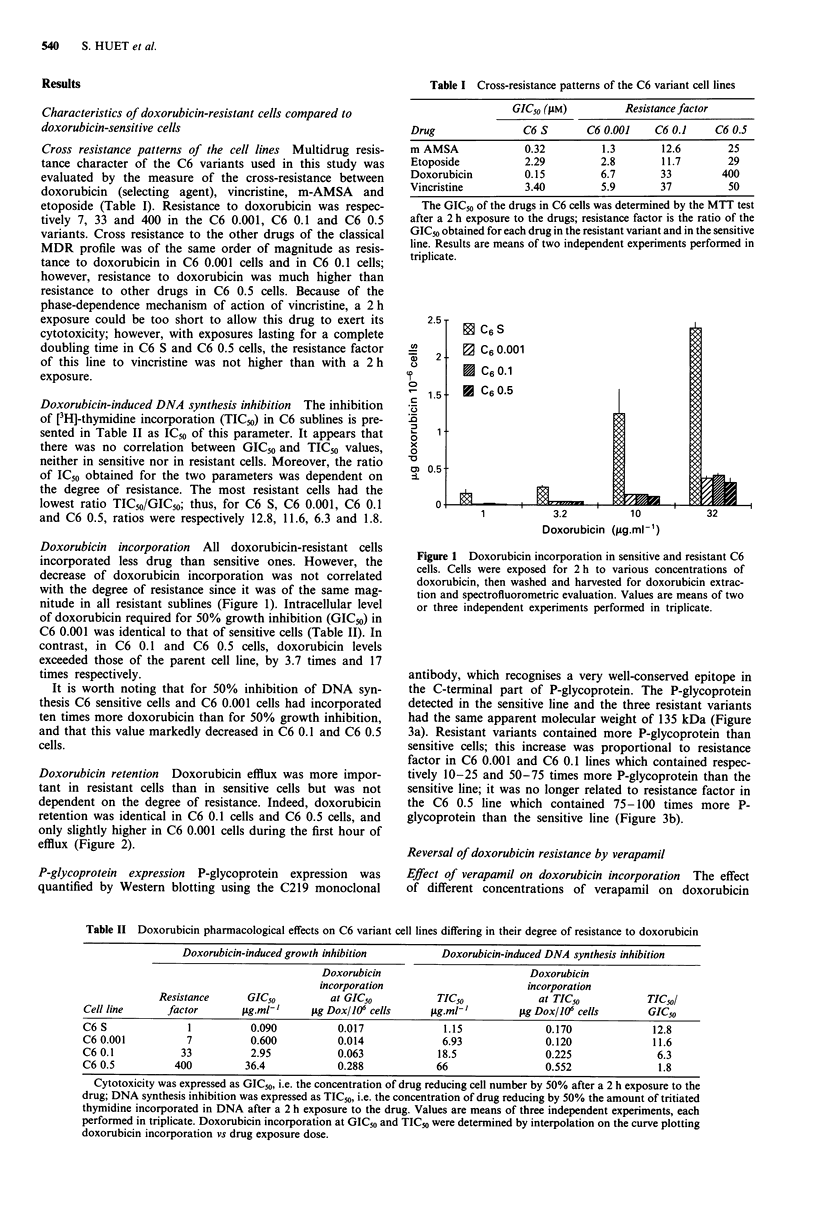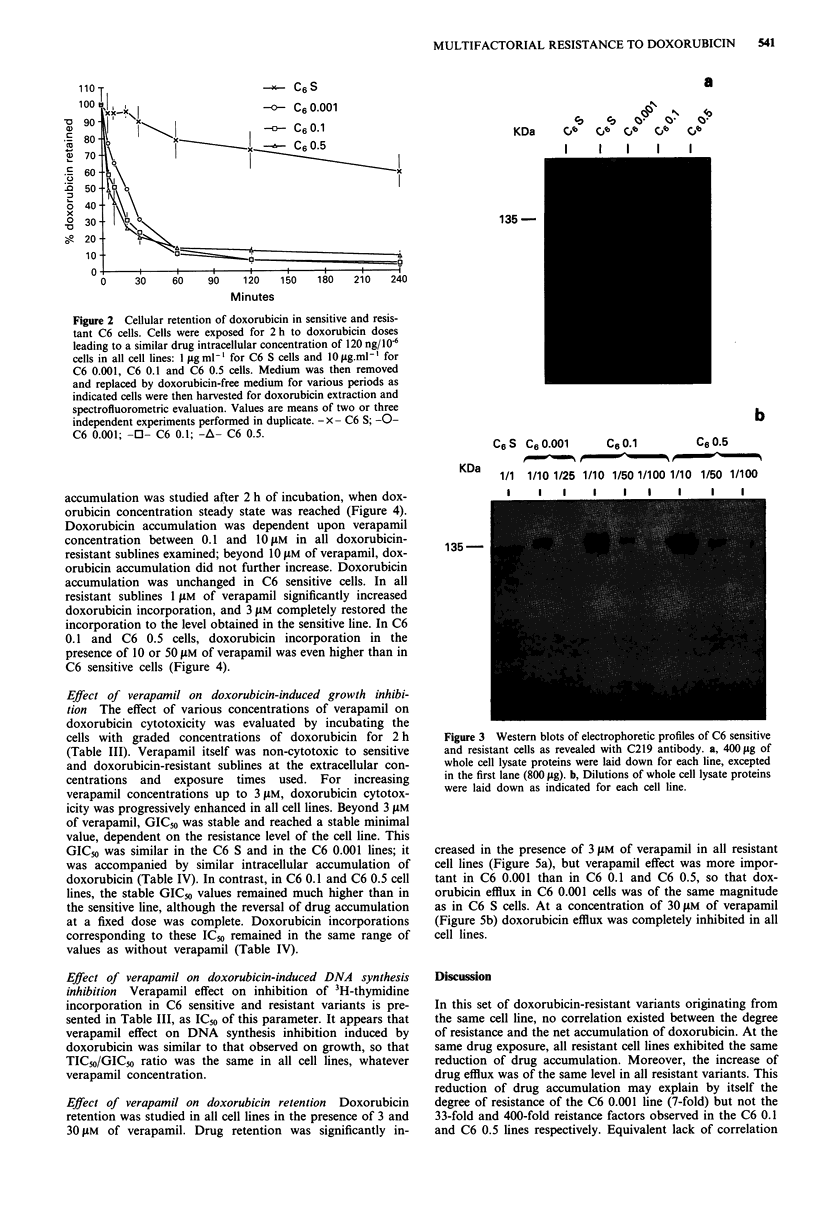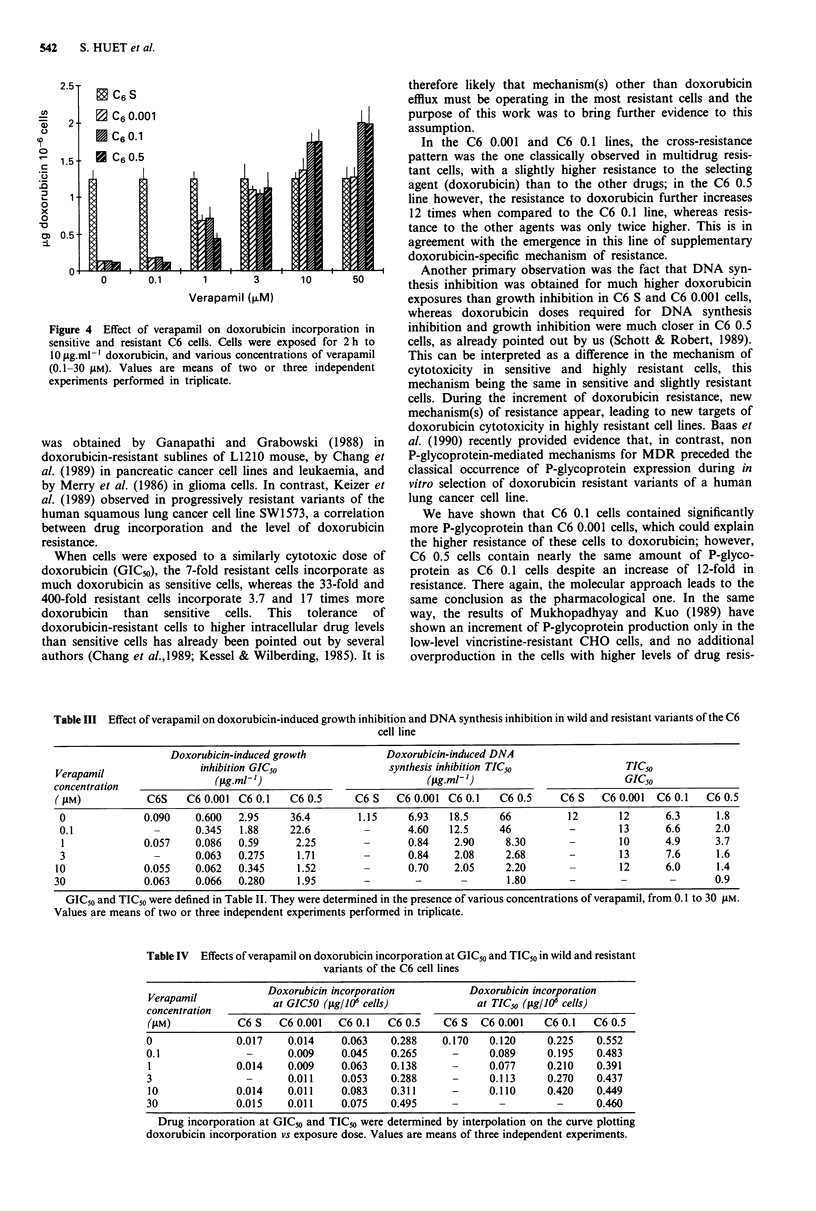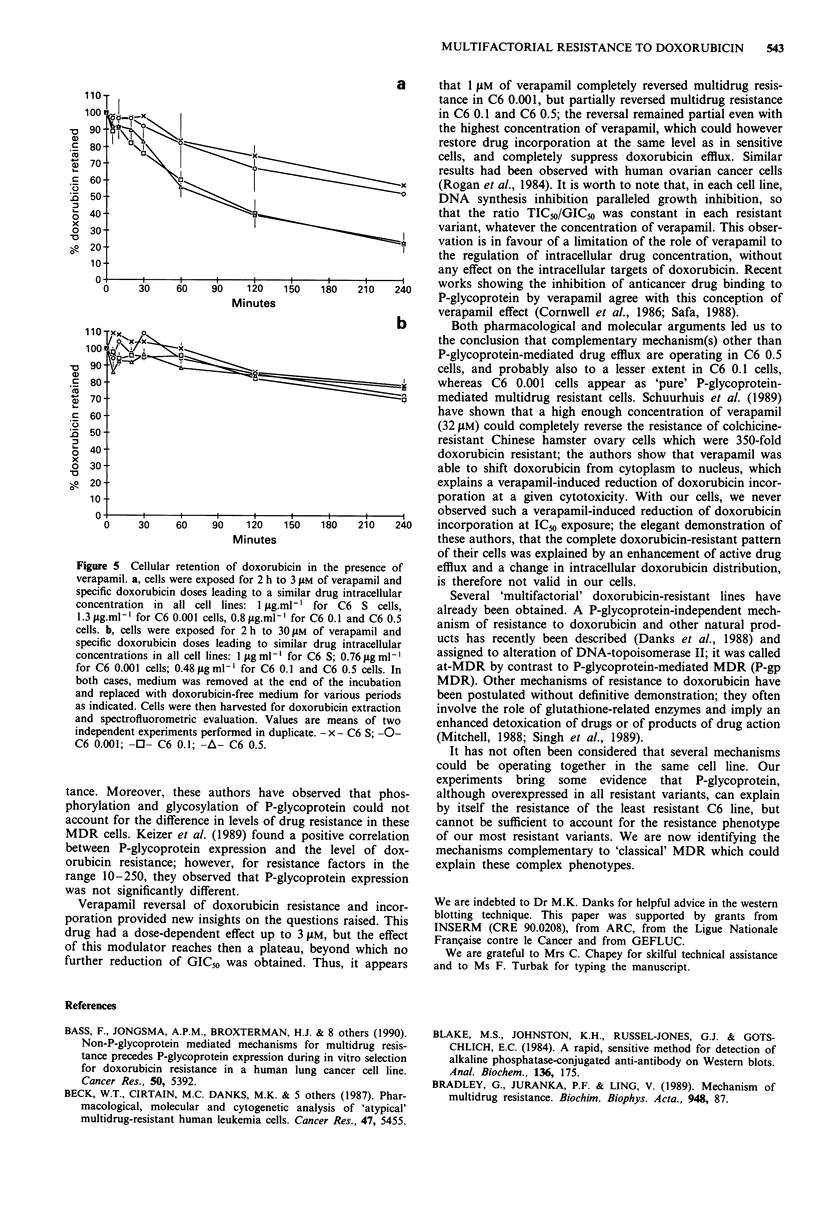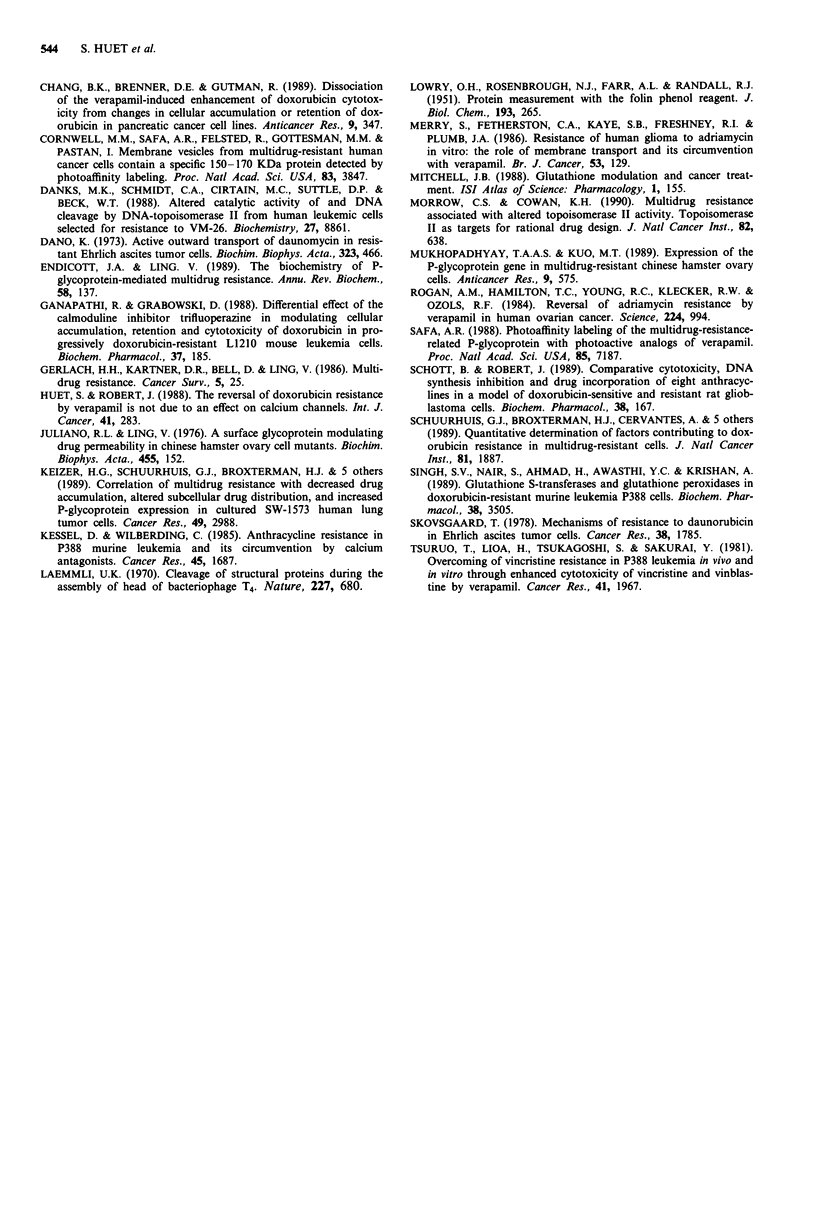# P-glycoprotein overexpression cannot explain the complete doxorubicin-resistance phenotype in rat glioblastoma cell lines.

**DOI:** 10.1038/bjc.1992.111

**Published:** 1992-04

**Authors:** S. Huet, B. Schott, J. Robert

**Affiliations:** Fondation Bergonié, Bordeaux, France.

## Abstract

**Images:**


*Br. J. Cancer* (1992), **65**, 538–544 © Macmillan Press Ltd., 1992

### P-glycoprotein overexpression cannot explain the complete doxorubicin-resistance phenotype in rat glioblastoma cell lines

S. Huet, B. Schott & J. Robert

*Fondation Bergonié and Université de Bordeaux II 180, rue de Saint-Genès - 33076 - Bordeaux Cedex, France.*

**Summary** We have associated pharmacological studies to a semi-quantitative evaluation of P-glycoprotein(s) expression, to establish if classical multidrug resistance (MDR) could account for the complete resistance phenotype exhibited by progressively doxorubicin-resistant rat glioblastoma cells. Three resistant variants (C6 0.001, C6 0.1 and C6 0.5) of the C6 glioblastoma cell line (C6 S) were selected by long-term culture in the presence of three concentrations of doxorubicin (0.001, 0.1 and 0.5 $\mu g.ml^{-1}$ respectively). The degree of doxorubicin resistance was respectively 7, 33 and 400, and all the cell variants were cross-resistant to m-AMSA, etoposide and vincristine. Doxorubicin incorporation was reduced similarly in all resistant cells, irrespective of the level of resistance. When exposed to their respective doxorubicin $IC_{50}$, the 7-fold resistant cells had the same intracellular drug incorporation as the sensitive cells, whereas the 33-fold and 400-fold resistant cells could incorporate respectively 3.7 and 17 times more drug. The ratio of doxorubicin exposures required for 50% DNA synthesis inhibition and 50% growth inhibition was dependent on the degree of resistance; this ratio was 12.8 in C6 S, 11.6 in C6 0.001, 6.3 in C6 0.1 and 1.8 in C6 0.5. P-glycoprotein(s) overexpression was of the same magnitude as the resistance factor in variants C6 0.001 and C6 0.1, but was lower than resistance factor in variant C6 0.5. Reversal of drug incorporation by verapamil was complete in all resistant cell lines; however, reversal of doxorubicin cytotoxicity was complete only in the 7-fold resistant line and was only partial in the most resistant lines, which remained 10-fold and 20-fold resistant to doxorubicin. These results suggest that classical MDR was the first phenotype selected by doxorubicin in C6 0.001, whereas mechanism(s) of doxorubicin resistance other than classical MDR are added in the most resistant lines.

Emergence of multidrug-resistant (MDR) tumour cells during treatment is one of the major problems in cancer chemotherapy. This resistance has been studied using drug-resistant cell lines as an experimental model of this phenomenon. Multidrug resistance is characterised by cross-resistance to functionally and structurally unrelated drugs (Skovsgaard, 1978) and by an impaired drug accumulation as compared to the sensitive parental cell lines (Dano, 1973). Concommitantly, overexpression of a high molecular weight membrane glycoprotein (P-glycoprotein), encoded by the *mdr*1 gene, is usually observed with the emergence of multidrug resistance (Juliano & Ling, 1976). Decreased drug retention has been attributed mainly to enhanced active efflux of the drug out of the cells, which is thought to occur via P-glycoprotein (Gerlach *et al.*, 1986) (see for review Bradley *et al.*, 1989; Endicott & Ling, 1989).

Multidrug-resistance can be reversed to various extents by treatment of cells with the calcium channel blocker verapamil (Tsuruo *et al.*, 1981). It is now clear that verapamil exerts its action by a mechanism that does not involve voltage-gated calcium channels (Huet & Robert, 1988). However, the exact mechanism of this effect is still unknown. The circumvention of resistance is associated with increased drug accumulation, probably through interaction of verapamil with the outward drug transporter of resistant cells, P-glycoprotein. Data demonstrating that verapamil inhibits the vinblastine photoaffinity labelling of P-glycoprotein suggest that interaction of this agent with P-glycoprotein could be the biochemical basis for its pharmacological effects in MDR cells (Cornwell *et al.*, 1986). Moreover, using photoaffinity analogs of verapamil, Safa (1988) has shown that P-glycoprotein in MDR cells is a specific acceptor for verapamil.

The relationship between drug incorporation and cytotoxicity of anthracyclines has been extensively studied with different approaches. In glioblastoma cells with high level of resistance, we have shown that resistant cells can tolerate higher amounts of drug than sensitive cells for a similar cytotoxicity (Schott & Robert, 1989). It had been also observed with this model that DNA synthesis inhibition occurred for higher doses than growth inhibition only in sensitive cells, suggesting that a distinct mechanism of cell death occurred in sensitive and resistant cells. Such features are not explained by the simple conception of P-glycoprotein as the unique mechanism for multidrug resistance, and suggest that additional mechanism(s) may lead to multidrug resistance.

Alternative forms of multidrug resistance became apparent in recent studies, especially a mechanism associated with alterations of DNA-topoisomerase II. This particular resistance was named by W.T. Beck 'atypical' MDR. In contrast to 'classical' P-glycoprotein-mediated MDR, cells expressing atypical MDR have no alteration in drug accumulation and retention, and do not overexpress P-glycoprotein (Beck *et al.*, 1987; Morrow & Cowan, 1990).

Using pharmacological and molecular approaches, we show in this paper that the mechanisms underlying resistance were dependent on the degree of resistance in a model of progressively doxorubicin-resistant rodent cells. Cells with low level of resistance did not respond similarly to doxorubicin and verapamil as cells with high level of resistance. Moreover, P-glycoprotein expression in these cell lines was only slightly different despite their different levels of resistance.

### Materials and methods

### *Drugs and materials*

Doxorubicin was a generous gift from Laboratoire Roger Bellon. Verapamil was clinical formulation (Isoptine®). Media and sera for cell culture originated from Seromed, Petri dishes from Nunc.

[$^3$H-methyl]thymidine was purchased from Amersham. Liquid scintillation medium Pico-fluor was provided by Packard.

C219 monoclonal antibody was purchased from Centocor through CIS-International.

Correspondence: J. Robert, Fondation Bergonié, Laboratoire de Biochimie, 180 rue de Saint-Genès - 33076 Bordeaux Cedex, France.
Received 6 September 1991; and in revised form 5 December 1991.

*Cell and culture conditions*

The C6 clone originated from a brain tumour induced in rat by N-methylnitrosourea. The resistant lines were obtained by exposure of the sensitive cells (C6 S) to stepwise increasing amounts of doxorubicin. Three levels of resistance: C6 0.001, C6 0.1 and C6 0.5 were selected; they could be characterised by the level of doxorubicin routinely tolerated by the cells in the culture medium: 0.001 $\mu g.ml^{-1}$, 0.1 $\mu g.ml^{-1}$, 0.5 $\mu g.ml^{-1}$ respectively. The cells were cultivated as monolayers in Petri dishes with Dulbecco's modified Eagle medium supplemented with 10% foetal calf serum and antibiotic mixture, at 37°, in a humidified atmosphere containing 5% $CO_2$. The cultures were replicated each week and the medium was changed each 2 of 3 days, depending on the cell density.

*Doxorubicin incorporation and [$^3$H-methyl]thymidine incorporation*

For these evaluations, $8.10^4$ C6 S cells or $10.10^4$ C6 0.001 cells were seeded in 10 $cm^2$ Petri dishes with 3 ml medium, or $25.10^4$ C6 0.1 cells or $50.10^4$ C6 0.5 cells were seeded in 20 $cm^2$ Petri dishes with 5 ml of medium without drug. The medium was changed 3 days later; on the fourth day, the number of cells reached approximately $2.10^6$ cells per dish in all cases. Drug incorporation was measured by substituting the medium by 3 ml new medium containing various concentrations of drug (0.032–320 $\mu g.ml^{-1}$) and the dishes were incubated at 37° for 2 h. One hour before the end of the drug exposure, 1 $\mu$Ci of [$^3$H-methyl]thymidine per dish was added. Then, the monolayers were washed twice with 0.15 M NaCl, harvested after gentle stirring and pelleted at 3,000 r.p.m. for 5 min. These steps were rapidly performed in order to avoid any efflux of the drug; 0.5 ml of water and 0.5 ml of 40% trichloracetic acid were successively added and the samples were kept at 4°C overnight, then centrifuged for 30 min at 3,000 r.p.m. The acid-soluble fraction was used to evaluate the intracellular concentration of non-covalently bound drug by fluorometry (spectrofluorometer SFM25 Kontron) with excitation and emission wavelengths set at 468 nm and 592 nm respectively. The acid-insoluble pellet was solubilised with 1 M NaOH and used to evaluate both the protein content (Lowry *et al.*, 1951) and the $^3$H radioactivity in a Beckman LS 1207 liquid scintillation spectrometer.

All incubations were performed in triplicate and three independent experiments were performed. In all cases, incorporation of [$^3$H-methyl]thymidine was referred to controls realised in the same conditions and incubated without drug. It was possible to define a $TIC_{50}$ value, i.e. the concentration of drug providing a 50% decrease of [$^3$H-methyl]thymidine incorporation.

*MTT assay*

A colorimetric assay utilising the tetrazolium salt, MTT, was used to assess cytotoxicity of a 2 h exposure to doxorubicin or other drugs in the presence or absence of verapamil. Cells in log phase were harvested from flasks using trypsin-EDTA. After resuspension in fresh medium, the cells were plated in a volume of 200 $\mu$l at $5.10^2$ cells per well for C6 S, $8.10^2$ cells per well for C6 0.001, $15.10^2$ cells per well for C6 0.1, and $20.10^2$ cells for C6 0.5. These cell densities were chosen because they allowed exponential growth throughout a 5 day period for C6 S and C6 0.001, and a 7 day period for C6 0.1 and C6 0.5. The plates were incubated at 37°C, in a humidified atmosphere containing 5% $CO_2$, during 1 day for C6 S and C6 0.001, and 2 days for C6 0.1 and C6 0.5 because their growth was slower. Then, culture medium was replaced by fresh medium containing doxorubicin or other drugs, and eventually verapamil, at the appropriate concentrations, and incubations were performed for 2 h at 37°C. After this drug exposure, monolayers were washed twice with culture medium followed by addition of 300 $\mu$l of drug-free medium per well. The plates were then incubated for 4 days (C6 S cells and C6 0.001 cells) and 5 days (C6 0.1 and C6 0.5). Thereafter, 300 $\mu$l of medium containing 0.5 $mg.ml^{-1}$ MTT were added in each well and the plate incubated at 37°C for additional 4 h. Medium was then removed from each well, 200 $\mu$l of DMSO were added and the plate shaken 5 min; absorbance was immediately determined on a two-wavelength microplate Auto reader (Biotek Instruments EL311) at test and reference wavelengths of 570 nm and 630 nm respectively. The precision of this method using triplicate determination is 10% (s.d.). The cytotoxicity was expressed as the $GIC_{50}$, i.e. the concentration of doxorubicin causing 50% decrease of absorbance as compared to controls incubated simultaneously without doxorubicin. Additional blank controls consisting in the same growth medium and drug conditions without cells were substracted from sample absorbance values.

*Doxorubicin efflux*

Different concentrations of extracellular doxorubicin were utilised in order to compensate for differences in cellular accumulation of doxorubicin between the sensitive and resistant sublines treated with or without verapamil. C6 S cells were pretreated with 1 $\mu g.ml^{-1}$ doxorubicin in all conditions; C6 0.001, C6 0.1 and C6 0.5 were pretreated with 10 $\mu g.ml^{-1}$ doxorubicin without verapamil; in the presence of 3 $\mu$M verapamil, C6 0.001, C6 0.1 and C6 0.5 were pretreated with 1.3, 0.8 and 0.8 $\mu g\ ml^{-1}$ doxorubicin respectively, and in the presence of 30 $\mu$M verapamil, C6 0.001, C6 0.1, and C6 0.5 were pretreated with 0.76, 0.48 and 0.48 $\mu g.ml^{-1}$ doxorubicin respectively. In these conditions, intracellular doxorubicin concentration was approximately 120 ng/$10^6$ cells in all cases.

After 2 h at 37°C, incubation medium was removed and monolayers were washed twice with NaCl 0.15 M and immediately incubated in an equal volume of fresh drug-free medium with or without verapamil at 37°C. At the prescribed times, Petri dishes were removed and processed as indicated for incorporation studies.

*Western blot analysis for P-glycoprotein*

Cells in log phase were washed twice with PBS, harvested from flasks by gentle stirring and pelleted at 1,500 r.p.m. for 5 min after counting. Cell pellets were washed with 40 mM Tris and pelleted at 2,000 r.p.m. for 5 min at 4°C. Supernatants were removed and replaced by 250 $\mu$l of 5 mM Tris, 6 mM $MgCl_2$, 1.5 $\mu$l Aprotinin (32.2 $TUI.ml^{-1}$). After 10 min incubation, cells were homogenised by sonication. We have checked with microscope that no intact cells remained. Then were added 250 $\mu$l 80 mM Tris, 6 mM $MgCl_2$, 1.5 $\mu$l Aprotinin, and 50 $\mu$l DNaseI; the mixture was left at room temperature for 30 min.

Protein measurement was done with Biorad reagent using bovine serum albumin as a standard. The samples were then frozen at −80°C.

Cell proteins, 800 $\mu$g to 200 $\mu$g per lane, were resolved by SDS-PAGE using the method of Laemmli (1970). Protein molecular weight standards were run in an adjacent lane, and proteins were localised on nitrocellulose by Coomassie blue staining. Proteins were transferred to nitrocellulose with an electroblotting buffer system (Milliblot SDE Millipore) by the method indicated by the manufacturer.

The blots were incubated 1 h at 37°C in blocking buffer (0.9% NaCl, 10 mM Tris-HCl pH 7.5, 0.02% sodium azide, 5% dry milk, 3% IgG free BSA, and 0.2% Tween 20), followed by incubation with C219 monoclonal antibody in fresh blocking buffer at 4°C overnight. The filters were washed with Tris-saline buffer, incubated with alkaline phosphatase-conjugated rabbit anti-mouse IgG in blocking buffer at room temperature for 1.5 h, rewashed with Tris-saline buffer and developed using BCIP substrate (0.5 $mg.ml^{-1}$) (Blake *et al.*, 1984).

Quantification was done by densitometric comparison of the spots obtained; diluting the extracts from resistant cells was done so as to obtain coloured spots of similar density as those obtained with an extract from sensitive cells.

### Results

### *Characteristics of doxorubicin-resistant cells compared to doxorubicin-sensitive cells*

*Cross resistance patterns of the cell lines* Multidrug resistance character of the C6 variants used in this study was evaluated by the measure of the cross-resistance between doxorubicin (selecting agent), vincristine, m-AMSA and etoposide (Table I). Resistance to doxorubicin was respectively 7, 33 and 400 in the C6 0.001, C6 0.1 and C6 0.5 variants. Cross resistance to the other drugs of the classical MDR profile was of the same order of magnitude as resistance to doxorubicin in C6 0.001 cells and in C6 0.1 cells; however, resistance to doxorubicin was much higher than resistance to other drugs in C6 0.5 cells. Because of the phase-dependence mechanism of action of vincristine, a 2 h exposure could be too short to allow this drug to exert its cytotoxicity; however, with exposures lasting for a complete doubling time in C6 S and C6 0.5 cells, the resistance factor of this line to vincristine was not higher than with a 2 h exposure.

*Doxorubicin-induced DNA synthesis inhibition* The inhibition of [$^3$H]-thymidine incorporation ($TIC_{50}$) in C6 sublines is presented in Table II as $IC_{50}$ of this parameter. It appears that there was no correlation between $GIC_{50}$ and $TIC_{50}$ values, neither in sensitive nor in resistant cells. Moreover, the ratio of $IC_{50}$ obtained for the two parameters was dependent on the degree of resistance. The most resistant cells had the lowest ratio $TIC_{50}/GIC_{50}$; thus, for C6 S, C6 0.001, C6 0.1 and C6 0.5, ratios were respectively 12.8, 11.6, 6.3 and 1.8.

*Doxorubicin incorporation* All doxorubicin-resistant cells incorporated less drug than sensitive ones. However, the decrease of doxorubicin incorporation was not correlated with the degree of resistance since it was of the same magnitude in all resistant sublines (Figure 1). Intracellular level of doxorubicin required for 50% growth inhibition ($GIC_{50}$) in C6 0.001 was identical to that of sensitive cells (Table II). In contrast, in C6 0.1 and C6 0.5 cells, doxorubicin levels exceeded those of the parent cell line, by 3.7 times and 17 times respectively.

It is worth noting that for 50% inhibition of DNA synthesis C6 sensitive cells and C6 0.001 cells had incorporated ten times more doxorubicin than for 50% growth inhibition, and that this value markedly decreased in C6 0.1 and C6 0.5 cells.

*Doxorubicin retention* Doxorubicin efflux was more important in resistant cells than in sensitive cells but was not dependent on the degree of resistance. Indeed, doxorubicin retention was identical in C6 0.1 cells and C6 0.5 cells, and only slightly higher in C6 0.001 cells during the first hour of efflux (Figure 2).

*P-glycoprotein expression* P-glycoprotein expression was quantified by Western blotting using the C219 monoclonal antibody, which recognises a very well-conserved epitope in the C-terminal part of P-glycoprotein. The P-glycoprotein detected in the sensitive line and the three resistant variants had the same apparent molecular weight of 135 kDa (Figure 3a). Resistant variants contained more P-glycoprotein than sensitive cells; this increase was proportional to resistance factor in C6 0.001 and C6 0.1 lines which contained respectively 10–25 and 50–75 times more P-glycoprotein than the sensitive line; it was no longer related to resistance factor in the C6 0.5 line which contained 75–100 times more P-glycoprotein than the sensitive line (Figure 3b).

**Table I** Cross-resistance patterns of the C6 variant cell lines

| | *$GIC_{50}$ (μM)* | *Resistance factor* | | |
|---|---|---|---|---|
| *Drug* | *C6 S* | *C6 0.001* | *C6 0.1* | *C6 0.5* |
| m AMSA | 0.32 | 1.3 | 12.6 | 25 |
| Etoposide | 2.29 | 2.8 | 11.7 | 29 |
| Doxorubicin | 0.15 | 6.7 | 33 | 400 |
| Vincristine | 3.40 | 5.9 | 37 | 50 |

The $GIC_{50}$ of the drugs in C6 cells was determined by the MTT test after a 2 h exposure to the drugs; resistance factor is the ratio of the $GIC_{50}$ obtained for each drug in the resistant variant and in the sensitive line. Results are means of two independent experiments performed in triplicate.

**Figure 1** Doxorubicin incorporation in sensitive and resistant C6 cells. Cells were exposed for 2 h to various concentrations of doxorubicin, then washed and harvested for doxorubicin extraction and spectrofluorometric evaluation. Values are means of two or three independent experiments performed in triplicate.

### *Reversal of doxorubicin resistance by verapamil*

*Effect of verapamil on doxorubicin incorporation* The effect of different concentrations of verapamil on doxorubicin

**Table II** Doxorubicin pharmacological effects on C6 variant cell lines differing in their degree of resistance to doxorubicin

| | *Doxorubicin-induced growth inhibition* | | | *Doxorubicin-induced DNA synthesis inhibition* | | |
|---|---|---|---|---|---|---|
| *Cell line* | *Resistance factor* | *$GIC_{50}$ μg.ml$^{-1}$* | *Doxorubicin incorporation at $GIC_{50}$ μg Dox/$10^6$ cells* | *$TIC_{50}$ μg.ml$^{-1}$* | *Doxorubicin incorporation at $TIC_{50}$ μg Dox/$10^6$ cells* | *$TIC_{50}$/ $GIC_{50}$* |
| C6 S | 1 | 0.090 | 0.017 | 1.15 | 0.170 | 12.8 |
| C6 0.001 | 7 | 0.600 | 0.014 | 6.93 | 0.120 | 11.6 |
| C6 0.1 | 33 | 2.95 | 0.063 | 18.5 | 0.225 | 6.3 |
| C6 0.5 | 400 | 36.4 | 0.288 | 66 | 0.552 | 1.8 |

Cytotoxicity was expressed as $GIC_{50}$, i.e. the concentration of drug reducing cell number by 50% after a 2 h exposure to the drug; DNA synthesis inhibition was expressed as $TIC_{50}$, i.e. the concentration of drug reducing by 50% the amount of tritiated thymidine incorporated in DNA after a 2 h exposure to the drug. Values are means of three independent experiments, each performed in triplicate. Doxorubicin incorporation at $GIC_{50}$ and $TIC_{50}$ were determined by interpolation on the curve plotting doxorubicin incorporation *vs* drug exposure dose.

**Figure 2** Cellular retention of doxorubicin in sensitive and resistant C6 cells. Cells were exposed for 2 h to doxorubicin doses leading to a similar drug intracellular concentration of 120 ng/$10^{-6}$ cells in all cell lines: 1 μg $ml^{-1}$ for C6 S cells and 10 μg.$ml^{-1}$ for C6 0.001, C6 0.1 and C6 0.5 cells. Medium was then removed and replaced by doxorubicin-free medium for various periods as indicated cells were then harvested for doxorubicin extraction and spectrofluorometric evaluation. Values are means of two or three independent experiments performed in duplicate. -×- C6 S; -O- C6 0.001; -□- C6 0.1; -Δ- C6 0.5.

**Figure 3** Western blots of electrophoretic profiles of C6 sensitive and resistant cells as revealed with C219 antibody. **a**, 400 μg of whole cell lysate proteins were laid down for each line, excepted in the first lane (800 μg). **b**, Dilutions of whole cell lysate proteins were laid down as indicated for each cell line.

accumulation was studied after 2 h of incubation, when doxorubicin concentration steady state was reached (Figure 4). Doxorubicin accumulation was dependent upon verapamil concentration between 0.1 and 10 μM in all doxorubicin-resistant sublines examined; beyond 10 μM of verapamil, doxorubicin accumulation did not further increase. Doxorubicin accumulation was unchanged in C6 sensitive cells. In all resistant sublines 1 μM of verapamil significantly increased doxorubicin incorporation, and 3 μM completely restored the incorporation to the level obtained in the sensitive line. In C6 0.1 and C6 0.5 cells, doxorubicin incorporation in the presence of 10 or 50 μM of verapamil was even higher than in C6 sensitive cells (Figure 4).

*Effect of verapamil on doxorubicin-induced growth inhibition* The effect of various concentrations of verapamil on doxorubicin cytotoxicity was evaluated by incubating the cells with graded concentrations of doxorubicin for 2 h (Table III). Verapamil itself was non-cytotoxic to sensitive and doxorubicin-resistant sublines at the extracellular concentrations and exposure times used. For increasing verapamil concentrations up to 3 μM, doxorubicin cytotoxicity was progressively enhanced in all cell lines. Beyond 3 μM of verapamil, $GIC_{50}$ was stable and reached a stable minimal value, dependent on the resistance level of the cell line. This $GIC_{50}$ was similar in the C6 S and in the C6 0.001 lines; it was accompanied by similar intracellular accumulation of doxorubicin (Table IV). In contrast, in C6 0.1 and C6 0.5 cell lines, the stable $GIC_{50}$ values remained much higher than in the sensitive line, although the reversal of drug accumulation at a fixed dose was complete. Doxorubicin incorporations corresponding to these $IC_{50}$ remained in the same range of values as without verapamil (Table IV).

*Effect of verapamil on doxorubicin-induced DNA synthesis inhibition* Verapamil effect on inhibition of $^3$H-thymidine incorporation in C6 sensitive and resistant variants is presented in Table III, as $IC_{50}$ of this parameter. It appears that verapamil effect on DNA synthesis inhibition induced by doxorubicin was similar to that observed on growth, so that $TIC_{50}/GIC_{50}$ ratio was the same in all cell lines, whatever verapamil concentration.

*Effect of verapamil on doxorubicin retention* Doxorubicin retention was studied in all cell lines in the presence of 3 and 30 μM of verapamil. Drug retention was significantly increased in the presence of 3 μM of verapamil in all resistant cell lines (Figure 5a), but verapamil effect was more important in C6 0.001 than in C6 0.1 and C6 0.5, so that doxorubicin efflux in C6 0.001 cells was of the same magnitude as in C6 S cells. At a concentration of 30 μM of verapamil (Figure 5b) doxorubicin efflux was completely inhibited in all cell lines.

### Discussion

In this set of doxorubicin-resistant variants originating from the same cell line, no correlation existed between the degree of resistance and the net accumulation of doxorubicin. At the same drug exposure, all resistant cell lines exhibited the same reduction of drug accumulation. Moreover, the increase of drug efflux was of the same level in all resistant variants. This reduction of drug accumulation may explain by itself the degree of resistance of the C6 0.001 line (7-fold) but not the 33-fold and 400-fold reistance factors observed in the C6 0.1 and C6 0.5 lines respectively. Equivalent lack of correlation

**Figure 4** Effect of verapamil on doxorubicin incorporation in sensitive and resistant C6 cells. Cells were exposed for 2 h to 10 $\mu g.ml^{-1}$ doxorubicin, and various concentrations of verapamil (0.1–30 µM). Values are means of two or three independent experiments performed in triplicate.

was obtained by Ganapathi and Grabowski (1988) in doxorubicin-resistant sublines of L1210 mouse, by Chang *et al.* (1989) in pancreatic cancer cell lines and leukaemia, and by Merry *et al.* (1986) in glioma cells. In contrast, Keizer *et al.* (1989) observed in progressively resistant variants of the human squamous lung cancer cell line SW1573, a correlation between drug incorporation and the level of doxorubicin resistance.

When cells were exposed to a similarly cytotoxic dose of doxorubicin ($GIC_{50}$), the 7-fold resistant cells incorporate as much doxorubicin as sensitive cells, whereas the 33-fold and 400-fold resistant cells incorporate 3.7 and 17 times more doxorubicin than sensitive cells. This tolerance of doxorubicin-resistant cells to higher intracellular drug levels than sensitive cells has already been pointed out by several authors (Chang *et al.*,1989; Kessel & Wilberding, 1985). It is therefore likely that mechanism(s) other than doxorubicin efflux must be operating in the most resistant cells and the purpose of this work was to bring further evidence to this assumption.

In the C6 0.001 and C6 0.1 lines, the cross-resistance pattern was the one classically observed in multidrug resistant cells, with a slightly higher resistance to the selecting agent (doxorubicin) than to the other drugs; in the C6 0.5 line however, the resistance to doxorubicin further increases 12 times when compared to the C6 0.1 line, whereas resistance to the other agents was only twice higher. This is in agreement with the emergence in this line of supplementary doxorubicin-specific mechanism of resistance.

Another primary observation was the fact that DNA synthesis inhibition was obtained for much higher doxorubicin exposures than growth inhibition in C6 S and C6 0.001 cells, whereas doxorubicin doses required for DNA synthesis inhibition and growth inhibition were much closer in C6 0.5 cells, as already pointed out by us (Schott & Robert, 1989). This can be interpreted as a difference in the mechanism of cytotoxicity in sensitive and highly resistant cells, this mechanism being the same in sensitive and slightly resistant cells. During the increment of doxorubicin resistance, new mechanism(s) of resistance appear, leading to new targets of doxorubicin cytotoxicity in highly resistant cell lines. Baas *et al.* (1990) recently provided evidence that, in contrast, non P-glycoprotein-mediated mechanisms for MDR preceded the classical occurrence of P-glycoprotein expression during *in vitro* selection of doxorubicin resistant variants of a human lung cancer cell line.

We have shown that C6 0.1 cells contained significantly more P-glycoprotein than C6 0.001 cells, which could explain the higher resistance of these cells to doxorubicin; however, C6 0.5 cells contain nearly the same amount of P-glycoprotein as C6 0.1 cells despite an increase of 12-fold in resistance. There again, the molecular approach leads to the same conclusion as the pharmacological one. In the same way, the results of Mukhopadhyay and Kuo (1989) have shown an increment of P-glycoprotein production only in the low-level vincristine-resistant CHO cells, and no additional overproduction in the cells with higher levels of drug resis-

**Table III** Effect of verapamil on doxorubicin-induced growth inhibition and DNA synthesis inhibition in wild and resistant variants of the C6 cell line

| *Verapamil concentration* | *Doxorubicin-induced growth inhibition $GIC_{50}$ ($\mu g.ml^{-1}$)* | | | | *Doxorubicin-induced DNA synthesis inhibition $TIC_{50}$ ($\mu g.ml^{-1}$)* | | | | *$TIC_{50}$ / $GIC_{50}$* | | | |
|---|---|---|---|---|---|---|---|---|---|---|---|---|
| *(µM)* | C6S | C6 0.001 | C6 0.1 | C6 0.5 | C6 S | C6 0.001 | C6 0.1 | C6 0.5 | C6 S | C6 0.001 | C6 0.1 | C6 0.5 |
| 0 | 0.090 | 0.600 | 2.95 | 36.4 | 1.15 | 6.93 | 18.5 | 66 | 12 | 12 | 6.3 | 1.8 |
| 0.1 | – | 0.345 | 1.88 | 22.6 | – | 4.60 | 12.5 | 46 | – | 13 | 6.6 | 2.0 |
| 1 | 0.057 | 0.086 | 0.59 | 2.25 | – | 0.84 | 2.90 | 8.30 | – | 10 | 4.9 | 3.7 |
| 3 | – | 0.063 | 0.275 | 1.71 | – | 0.84 | 2.08 | 2.68 | – | 13 | 7.6 | 1.6 |
| 10 | 0.055 | 0.062 | 0.345 | 1.52 | – | 0.70 | 2.05 | 2.20 | – | 12 | 6.0 | 1.4 |
| 30 | 0.063 | 0.066 | 0.280 | 1.95 | – | – | – | 1.80 | – | – | – | 0.9 |

$GIC_{50}$ and $TIC_{50}$ were defined in Table II. They were determined in the presence of various concentrations of verapamil, from 0.1 to 30 µM. Values are means of two or three independent experiments performed in triplicate.

**Table IV** Effects of verapamil on doxorubicin incorporation at $GIC_{50}$ and $TIC_{50}$ in wild and resistant variants of the C6 cell lines

| *Verapamil concentration* | *Doxorubicin incorporation at GIC50 (µg/10⁶ cells)* | | | | *Doxorubicin incorporation at $TIC_{50}$ (µg/10⁶ cells)* | | | |
|---|---|---|---|---|---|---|---|---|
| *(µM)* | C6 S | C6 0.001 | C6 0.1 | C6 0.5 | C6 S | C6 0.001 | C6 0.1 | C6 0.5 |
| 0 | 0.017 | 0.014 | 0.063 | 0.288 | 0.170 | 0.120 | 0.225 | 0.552 |
| 0.1 | – | 0.009 | 0.045 | 0.265 | – | 0.089 | 0.195 | 0.483 |
| 1 | 0.014 | 0.009 | 0.063 | 0.138 | – | 0.077 | 0.210 | 0.391 |
| 3 | – | 0.011 | 0.053 | 0.288 | – | 0.113 | 0.270 | 0.437 |
| 10 | 0.014 | 0.011 | 0.083 | 0.311 | – | 0.110 | 0.420 | 0.449 |
| 30 | 0.015 | 0.011 | 0.075 | 0.495 | – | – | – | 0.460 |

Drug incorporation at $GIC_{50}$ and $TIC_{50}$ were determined by interpolation on the curve plotting doxorubicin incorporation *vs* exposure dose. Values are means of three independent experiments.

**Figure 5** Cellular retention of doxorubicin in the presence of verapamil. **a**, cells were exposed for 2 h to 3 μM of verapamil and specific doxorubicin doses leading to a similar drug intracellular concentration in all cell lines: 1 μg.ml$^{-1}$ for C6 S cells, 1.3 μg.ml$^{-1}$ for C6 0.001 cells, 0.8 μg.ml$^{-1}$ for C6 0.1 and C6 0.5 cells. **b**, cells were exposed for 2 h to 30 μM of verapamil and specific doxorubicin doses leading to similar drug intracellular concentrations in all cell lines: 1 μg ml$^{-1}$ for C6 S; 0.76 μg ml$^{-1}$ for C6 0.001 cells; 0.48 μg ml$^{-1}$ for C6 0.1 and C6 0.5 cells. In both cases, medium was removed at the end of the incubation and replaced with doxorubicin-free medium for various periods as indicated. Cells were then harvested for doxorubicin extraction and spectrofluorometric evaluation. Values are means of two independent experiments performed in duplicate. -×- C6 S; -O- C6 0.001; -□- C6 0.1; -Δ- C6 0.5.

tance. Moreover, these authors have observed that phosphorylation and glycosylation of P-glycoprotein could not account for the difference in levels of drug resistance in these MDR cells. Keizer *et al.* (1989) found a positive correlation between P-glycoprotein expression and the level of doxorubicin resistance; however, for resistance factors in the range 10–250, they observed that P-glycoprotein expression was not significantly different.

Verapamil reversal of doxorubicin resistance and incorporation provided new insights on the questions raised. This drug had a dose-dependent effect up to 3 μM, but the effect of this modulator reaches then a plateau, beyond which no further reduction of $GIC_{50}$ was obtained. Thus, it appears that 1 μM of verapamil completely reversed multidrug resistance in C6 0.001, but partially reversed multidrug resistance in C6 0.1 and C6 0.5; the reversal remained partial even with the highest concentration of verapamil, which could however restore drug incorporation at the same level as in sensitive cells, and completely suppress doxorubicin efflux. Similar results had been observed with human ovarian cancer cells (Rogan *et al.*, 1984). It is worth to note that, in each cell line, DNA synthesis inhibition paralleled growth inhibition, so that the ratio $TIC_{50}/GIC_{50}$ was constant in each resistant variant, whatever the concentration of verapamil. This observation is in favour of a limitation of the role of verapamil to the regulation of intracellular drug concentration, without any effect on the intracellular targets of doxorubicin. Recent works showing the inhibition of anticancer drug binding to P-glycoprotein by verapamil agree with this conception of verapamil effect (Cornwell *et al.*, 1986; Safa, 1988).

Both pharmacological and molecular arguments led us to the conclusion that complementary mechanism(s) other than P-glycoprotein-mediated drug efflux are operating in C6 0.5 cells, and probably also to a lesser extent in C6 0.1 cells, whereas C6 0.001 cells appear as 'pure' P-glycoprotein-mediated multidrug resistant cells. Schuurhuis *et al.* (1989) have shown that a high enough concentration of verapamil (32 μM) could completely reverse the resistance of colchicine-resistant Chinese hamster ovary cells which were 350-fold doxorubicin resistant; the authors show that verapamil was able to shift doxorubicin from cytoplasm to nucleus, which explains a verapamil-induced reduction of doxorubicin incorporation at a given cytotoxicity. With our cells, we never observed such a verapamil-induced reduction of doxorubicin incorporation at $IC_{50}$ exposure; the elegant demonstration of these authors, that the complete doxorubicin-resistant pattern of their cells was explained by an enhancement of active drug efflux and a change in intracellular doxorubicin distribution, is therefore not valid in our cells.

Several 'multifactorial' doxorubicin-resistant lines have already been obtained. A P-glycoprotein-independent mechanism of resistance to doxorubicin and other natural products has recently been described (Danks *et al.*, 1988) and assigned to alteration of DNA-topoisomerase II; it was called at-MDR by contrast to P-glycoprotein-mediated MDR (P-gp MDR). Other mechanisms of resistance to doxorubicin have been postulated without definitive demonstration; they often involve the role of glutathione-related enzymes and imply an enhanced detoxication of drugs or of products of drug action (Mitchell, 1988; Singh *et al.*, 1989).

It has not often been considered that several mechanisms could be operating together in the same cell line. Our experiments bring some evidence that P-glycoprotein, although overexpressed in all resistant variants, can explain by itself the resistance of the least resistant C6 line, but cannot be sufficient to account for the resistance phenotype of our most resistant variants. We are now identifying the mechanisms complementary to 'classical' MDR which could explain these complex phenotypes.

We are indebted to Dr M.K. Danks for helpful advice in the western blotting technique. This paper was supported by grants from INSERM (CRE 90.0208), from ARC, from the Ligue Nationale Française contre le Cancer and from GEFLUC.

We are grateful to Mrs C. Chapey for skilful technical assistance and to Ms F. Turbak for typing the manuscript.

**References**

BASS, F., JONGSMA, A.P.M., BROXTERMAN, H.J. & 8 others (1990). Non-P-glycoprotein mediated mechanisms for multidrug resistance precedes P-glycoprotein expression during in vitro selection for doxorubicin resistance in a human lung cancer cell line. *Cancer Res.*, **50**, 5392.

BECK, W.T., CIRTAIN, M.C. DANKS, M.K. & 5 others (1987). Pharmacological, molecular and cytogenetic analysis of 'atypical' multidrug-resistant human leukemia cells. *Cancer Res.*, **47**, 5455.

BLAKE, M.S., JOHNSTON, K.H., RUSSEL-JONES, G.J. & GOTSCHLICH, E.C. (1984). A rapid, sensitive method for detection of alkaline phosphatase-conjugated anti-antibody on Western blots. *Anal. Biochem.*, **136**, 175.

BRADLEY, G., JURANKA, P.F. & LING, V. (1989). Mechanism of multidrug resistance. *Biochim. Biophys. Acta.*, **948**, 87.

CHANG, B.K., BRENNER, D.E. & GUTMAN, R. (1989). Dissociation of the verapamil-induced enhancement of doxorubicin cytotoxicity from changes in cellular accumulation or retention of doxorubicin in pancreatic cancer cell lines. *Anticancer Res.*, **9**, 347.

CORNWELL, M.M., SAFA, A.R., FELSTED, R., GOTTESMAN, M.M. & PASTAN, I. Membrane vesicles from multidrug-resistant human cancer cells contain a specific 150–170 KDa protein detected by photoaffinity labeling. *Proc. Natl Acad. Sci. USA*, **83**, 3847.

DANKS, M.K., SCHMIDT, C.A., CIRTAIN, M.C., SUTTLE, D.P. & BECK, W.T. (1988). Altered catalytic activity of and DNA cleavage by DNA-topoisomerase II from human leukemic cells selected for resistance to VM-26. *Biochemistry*, **27**, 8861.

DANO, K. (1973). Active outward transport of daunomycin in resistant Ehrlich ascites tumor cells. *Biochim. Biophys. Acta.*, **323**, 466.

ENDICOTT, J.A. & LING. V. (1989). The biochemistry of P-glycoprotein-mediated multidrug resistance. *Annu. Rev. Biochem.*, **58**, 137.

GANAPATHI, R. & GRABOWSKI, D. (1988). Differential effect of the calmoduline inhibitor trifluoperazine in modulating cellular accumulation, retention and cytotoxicity of doxorubicin in progressively doxorubicin-resistant L1210 mouse leukemia cells. *Biochem. Pharmacol.*, **37**, 185.

GERLACH, H.H., KARTNER, D.R., BELL, D. & LING, V. (1986). Multidrug resistance. *Cancer Surv.*, **5**, 25.

HUET, S. & ROBERT, J. (1988). The reversal of doxorubicin resistance by verapamil is not due to an effect on calcium channels. *Int. J. Cancer*, **41**, 283.

JULIANO, R.L. & LING, V. (1976). A surface glycoprotein modulating drug permeability in chinese hamster ovary cell mutants. *Biochim. Biophys. Acta.*, **455**, 152.

KEIZER, H.G., SCHUURHUIS, G.J., BROXTERMAN, H.J. & 5 others (1989). Correlation of multidrug resistance with decreased drug accumulation, altered subcellular drug distribution, and increased P-glycoprotein expression in cultured SW-1573 human lung tumor cells. *Cancer Res.*, **49**, 2988.

KESSEL, D. & WILBERDING, C. (1985). Anthracycline resistance in P388 murine leukemia and its circumvention by calcium antagonists. *Cancer Res.*, **45**, 1687.

LAEMMLI, U.K. (1970). Cleavage of structural proteins during the assembly of head of bacteriophage $T_4$. *Nature*, **227**, 680.

LOWRY, O.H., ROSENBROUGH, N.J., FARR, A.L. & RANDALL, R.J. (1951). Protein measurement with the folin phenol reagent. *J. Biol. Chem.*, **193**, 265.

MERRY, S., FETHERSTON, C.A., KAYE, S.B., FRESHNEY, R.I. & PLUMB, J.A. (1986). Resistance of human glioma to adriamycin in vitro: the role of membrane transport and its circumvention with verapamil. *Br. J. Cancer*, **53**, 129.

MITCHELL, J.B. (1988). Glutathione modulation and cancer treatment. *ISI Atlas of Science: Pharmacology*, **1**, 155.

MORROW, C.S. & COWAN, K.H. (1990). Multidrug resistance associated with altered topoisomerase II activity. Topoisomerase II as targets for rational drug design. *J. Natl Cancer Inst.*, **82**, 638.

MUKHOPADHYAY, T.A.A.S. & KUO, M.T. (1989). Expression of the P-glycoprotein gene in multidrug-resistant chinese hamster ovary cells. *Anticancer Res.*, **9**, 575.

ROGAN, A.M., HAMILTON, T.C., YOUNG, R.C., KLECKER, R.W. & OZOLS, R.F. (1984). Reversal of adriamycin resistance by verapamil in human ovarian cancer. *Science*, **224**, 994.

SAFA, A.R. (1988). Photoaffinity labeling of the multidrug-resistance-related P-glycoprotein with photoactive analogs of verapamil. *Proc. Natl Acad. Sci. USA*, **85**, 7187.

SCHOTT, B. & ROBERT, J. (1989). Comparative cytotoxicity, DNA synthesis inhibition and drug incorporation of eight anthracyclines in a model of doxorubicin-sensitive and resistant rat glioblastoma cells. *Biochem. Pharmacol.*, **38**, 167.

SCHUURHUIS, G.J., BROXTERMAN, H.J., CERVANTES, A. & 5 others (1989). Quantitative determination of factors contributing to doxorubicin resistance in multidrug-resistant cells. *J. Natl Cancer Inst.*, **81**, 1887.

SINGH, S.V., NAIR, S., AHMAD, H., AWASTHI, Y.C. & KRISHAN, A. (1989). Glutathione S-transferases and glutathione peroxidases in doxorubicin-resistant murine leukemia P388 cells. *Biochem. Pharmacol.*, **38**, 3505.

SKOVSGAARD, T. (1978). Mechanisms of resistance to daunorubicin in Ehrlich ascites tumor cells. *Cancer Res.*, **38**, 1785.

TSURUO, T., LIOA, H., TSUKAGOSHI, S. & SAKURAI, Y. (1981). Overcoming of vincristine resistance in P388 leukemia *in vivo* and *in vitro* through enhanced cytotoxicity of vincristine and vinblastine by verapamil. *Cancer Res.*, **41**, 1967.